# Connections Between Diabetes Mellitus, Other Metabolic and Endocrine Dysfunctions and Cardiovascular Pathologies—Second Edition

**DOI:** 10.3390/biomedicines14010250

**Published:** 2026-01-22

**Authors:** Cristina Tudoran, Dragos Cozma

**Affiliations:** 1 Department VII, Internal Medicine II, Discipline of Cardiology, Victor Babeș University of Medicine and Pharmacy, E. Murgu Square, Nr. 2, 300041 Timisoara, Romania; 2 County Emergency Hospital “Pius Brinzeu”, L. Rebreanu, Nr. 156, 300723 Timisoara, Romania; 3 Center of Molecular Research in Nephrology and Vascular Disease, Faculty of the Victor Babeș University of Medicine and Pharmacy, E. Murgu Square, Nr. 2, 300041 Timisoara, Romania; 4 Institute of Cardiovascular Diseases Timisoara, 300310 Timisoara, Romania; dragos.cozma@umft.ro; 5 Cardiology Department, Victor Babeș University of Medicine and Pharmacy, 300041 Timisoara, Romania; 6 Research Center of the Institute of Cardiovascular Diseases Timisoara, 300310 Timisoara, Romania

The association between cardiovascular (CV) pathologies and metabolic/endocrine dysfunctions was first observed a long time ago, and great emphasis has been given to this relationship. Several guidelines of the European Society of Cardiology on coronary artery disease, arterial hypertension, heart failure (HF), and dyslipidemia include chapters on diabetes mellitus (DM) and chronic kidney disease (CKD) [1,2,3,4,5,6]. However, there are still unanswered questions regarding common pathophysiologic pathways, risk profile, and management of these comorbid associations of cardio-endocrine/metabolic pathologies. Another subject of interest is the benefits of newly developed drugs, and also their safety profiles.

In recent decades, the connections between endocrine dysfunctions and the CV system (CVS) have captivated the attention of several researchers [7,8]. Recent studies highlight that this morbid relationship begins even sooner than initially assumed. Besides genetic factors [9], multiple hormones (thyroid and sexual hormones, cortisol, insulin-like growth factor, etc.) influence the evolution of the CVS starting from the intrauterine evolution and play a pivotal role in its structural and functional development throughout childhood. On one side, hormones are involved in the maintenance of blood pressure, fluid balance, and myocardial remodelling, and, on the other side, endocrine imbalance may lead to CV alterations. Calcaterra et al. explored the heart’s intrinsic endocrine activity and how hormonal signals interact with the development of the CVS starting from the fetal state and continuing through early childhood [7]. Metabolic, thyroid, and other endocrine dysfunctions could lead to the earlier onset of CV pathologies in adult life [10]. The early interplay between the endocrine/metabolic system and CVS could account for the clustering of several metabolic and CV pathologies in the same patient, during adult life.

Another topic is the impact of sex-related differences on CV and metabolic pathologies. Stoiculescu et al. analyzed the influence of sex and DM on the rehospitalization rate in 1018 patients with HF with mildly reduced or/and preserved ejection fraction [11]. They determined that women without DM represent a low-risk phenotype, whereas diabetic men exhibit the highest risk of recurrent hospitalizations due to HF decompensation [11]. Metabolically-dysfunction-associated steatotic liver disease (MASLD) is common in patients with DM [12], and was for a long time considered to be more frequently diagnosed in men. Up-to-date research has demonstrated that, in women, MASLD often develops later, typically after menopause, being associated with a greater burden of comorbidities, higher mortality, increased cirrhosis prevalence, and a higher likelihood of requiring liver transplantation. New data, supported by detailed hepatic evaluations, suggest that postmenopausal women with visceral obesity showed similar or worse liver and cardiometabolic profiles than men, despite the failure of routine clinical and laboratory data (transaminases and the Fatty Liver Index) to diagnose MASLD. In women over 50 years old, anthropometric height-adjusted measurements (body mass index, waist circumference, waist-to-height ratio) and non-invasive markers of fibrosis and steatosis, together with the application of sex-specific cut-offs, may help reveal a comparable or slightly higher burden of disease associated with visceral fat accumulation. These data indicate that a more tailored approach, including sex-specific clinical and diagnostic criteria, for the detection of MASLD is needed in postmenopausal women, particularly in those with central obesity, to ensure early identification and management of this liver disease [13].

With the availability of modern tools for the diagnosis and management of HF, researchers realized that there is a bidirectional relationship between HF and metabolic/endocrine dysfunctions. As HF worsens, more and more organs are affected. Dilated cardiomyopathy (DCM) is often diagnosed in patients with severe signs and symptoms of HF. As a connection between metabolic/endocrine and CVD has been established, researchers are focusing on a possible correlation between type 2 DM (T2DM), obesity, thyroid dysfunction, and the prevalence of DCM [14]. In their retrospective cohort study on 1079 patients, Tica et al. [15] demonstrated that in non-insulin-treated T2DM patients, hypothyroidism and male sex were independently associated with an increased risk of developing DCM. These findings support the hypothesis that the coexistence of an increased metabolic burden (DM, obesity, metabolic syndrome), hypothyroidism, and even sex-related risk factors in the same patient group could favour a higher incidence of DCM or other CV alterations [16,17].

In recent years, researchers have observed the co-occurrence of multiple diseases in the same patient, which at first glance appear to have little in common. Another example of multiple cardio-metabolic dysfunctions and other peculiar diseases is the co-existence of DM, CV pathologies, and periodontal disease (PD) in the same individual [18,19,20]. The increased incidence of all three pathologies observed in recent years could partially explain this phenomenon. Another possible explanation could result from common pathophysiological mechanisms, such as chronic inflammation (elevated levels of Interleukin (IL)-6, tumour Necrosis Factor (TNF)-α, and reactive protein C (CRP)), immune dysregulation, oxidative stress, and microbial dysbiosis. DM exacerbates PD and accelerates tissue destruction via hyperglycaemia, while periodontitis worsens glycemic control and insulin resistance (IR). Both conditions independently elevate CVR. Periodontal therapy has led to lower glycemic levels and improved endothelial dysfunction, while cardiometabolic therapies (statins, Glucagon-Like Peptide (GLP-1) receptor agonists, Sodium-Glucose Cotransporter-2 inhibitors (SGLT2i)) ameliorated PD [20].

Peculiar adverse hormonal effects such as tertiary hyperparathyroidism (THPT), often encountered in CKD [21] due to prolonged secondary hyperparathyroidism, have been diagnosed in patients with diabetic nephropathy. THPT in DM favours vascular calcifications and increases the risk of developing major CV events. These patients raise diagnostic and therapeutic challenges, often requiring a multidisciplinary approach to address CV, renal, and metabolic pathologies [22]. The absence of standardized diagnostic criteria delays the diagnosis of THPT in patients with diabetic nephropathy, leading to inconsistent treatment strategies. Management must therefore integrate targeted metabolic and CV strategies, alongside specific therapies for THPT, including vitamin D analogues, calcimimetics, and phosphate-lowering drugs [22]. Patients with diabetic nephropathy and THPT undergoing haemodialysis are particularly difficult to treat as current guidelines offer wide reference ranges of optimal parathormone targets.

Referring to ESC guidelines, highly recommended HF therapies such as first-generation SGLT2i (empagliflozin and dapagliflozin) have substantially reshaped the management of HF and also of T2DM, owing to their glucose-lowering properties and to their consistent CV and renal protective effects [2,23,24]. The purpose of developing next-generation SGLT2-based therapies is to refine pharmacological selectivity, optimize pharmacokinetic profiles, and expand therapeutic applicability in patients without DM. Next-generation SGLT2is are characterized by structural and pharmacokinetic innovations, refined or deliberately balanced SGLT2/SGLT1 selectivity profiles, and enhanced engagement of extra-renal and glucose-independent pathways, including myocardial energy consumption, inflammatory modulation, vascular function, and gut–heart signalling. Dual SGLT1/SGLT2 inhibition further broadens therapeutic use by incorporating incretin-mediated mechanisms and intestinal glucose modulation, which may be particularly relevant in patients with high CV risk or with acute/decompensated CV pathologies. Recent findings indicate that the renal benefits of SGLT2i are disease-specific, with differential responses due to the underlying disease. Similarly, in HF, SGLT2-based therapies target both acute hemodynamic decompensation and chronic myocardial remodelling, supporting their use in HF [25].

Concerning risk reduction strategies, statins are widely used lipid-lowering agents that significantly reduce CV morbidity and mortality by lowering low-density lipoprotein (LDL) levels. Although employed for decades in patients with CVD, researchers suggested that they are prone to drug-to-drug interactions, and even some vitamins could influence their effects [26]. In their study, Cozma et al. explore the complex relationship between statins, vitamin D, and their impact on CV outcomes [27]. Both molecules intersect metabolically at 7-dehydrocholesterol. While theoretical concerns exist about the capacity of statins to impair vitamin D synthesis, clinical studies suggest a neutral or modestly positive effect on circulating 25(OH)D levels. Statin therapy does not appear to induce vitamin D deficiency and may modestly increase serum 25(OH)D concentrations, particularly lipophilic statins such as rosuvastatin and atorvastatin. Although routine vitamin D supplementation is not indicated in all statin-treated individuals, targeted repletion in those with confirmed deficiency—especially in the context of statin-associated muscle symptoms—may enhance tolerability and therapeutic adherence. While low vitamin D levels are associated with higher CV risk, supplementation has demonstrated consistent benefit in reducing major CV events, underlying the need for targeted research in high-risk, vitamin D-deficient populations [27].

Several studies demonstrated that hepatic overproduction of very-low-density lipoprotein (VLDL) worsens dyslipidemia and increases CV risk [28,29]. As the levels of cyclic adenosine monophosphate (cAMP) are indirectly correlated to the activation of the microsomal triglyceride transfer protein (MTP), which is essential in the hepatic production of VLDL, theophylline, a methylxanthine found in tea, may alter the hepatic production of VLDL by increasing intracellular cAMP. Park et al. demonstrated in their ex vivo rat model that tea-derived theophylline could directly inhibit MTP activity in rat hepatocytes after diet-induced increased MTP levels [30]. This inhibition decreases the secretion of triglycerides, total cholesterol, and VLDL while simultaneously doubling the high-density lipoprotein (HDL) output, thereby improving the TC/HDL-C and TG/HDL-C ratios and the atherogenic index. These results highlight the potential of theophylline to improve diet-induced dyslipidemia and reduce CV risk. Considering that theophylline is naturally abundant in common dietary sources, such as green tea, cacao, and kola nuts, its regular consumption may improve lipid profile and reduce the associated CV risk, allowing the reduction in lipid-lowering drug doses [30].

Future studies should focus on early identification and targeted management of CV risk factors including sex-specific hormonal dysregulation and refinement of CV risk-profiles strategies that address combined genetic, demographic, sexual, and metabolic characteristics [7,15]. Large-scale double-blind randomized studies, combined with multidisciplinary approaches and targeted health strategies, are required to address coexisting endocrine/metabolic, renal, and CV pathologies and to improve long-term outcomes for this category of very high-risk patients [20,22,25,27,30].

Regarding peculiar therapeutic options discussed in this reprint, such as the effects of theophylline on lipid metabolism [30], of vitamin D on statins [27], and the CV, renal, and metabolic effects of next-generation SGLT2i [25], future research should rigorously evaluate their effectiveness, optimal dosing regimens, possible adverse effects, and long-term safety in specific populations, such as patients with cardiomyopathies, liver diseases, advanced CKD, or acute hemodynamic instability, and the long-term implications of early initiation strategies in acute HF. Additional challenges relate to implementation, including cost-effectiveness, access, and integration into guideline-directed therapy without unintended adverse interactions [22,25,27,30].

## Data Availability

All of the data are presented in the manuscript.
